# Phenotypic Characterization of the Martina Franca Donkey: An Endangered Italian Donkey Breed

**DOI:** 10.3390/ani14131950

**Published:** 2024-07-01

**Authors:** Alberto De Berardinis, Roberta Bucci, Ippolito De Amicis, Francesca Del Signore, Salvatore Parrillo, Ivano Massirio, Massimo Vignoli, Augusto Carluccio

**Affiliations:** Department of Veterinary Medicine, University of Teramo, SP 18, 64100 Teramo, Italy; alberto.deberardinis@studenti.unite.it (A.D.B.); rbucci@unite.it (R.B.); ideamicis@unite.it (I.D.A.); sparrillo@unite.it (S.P.); imassirio@unite.it (I.M.); acarluccio@unite.it (A.C.)

**Keywords:** Martina Franca donkey morphology, endangered breed, phenotype, body measurements, Montanaro study

## Abstract

**Simple Summary:**

The Martina Franca (MF) donkey breed primarily inhabits rural areas in the Apulia region of Italy, and, to a lesser extent, small farms in northern and central Italy. This study examined 91 MF donkeys from eight breeding stations in central and southern Italy. We assessed 27 morphological features of the donkey using a tape measure, Hauptner’s hippometer, and digital animal scales. The MF Donkey and Murgese Horse Breeders’ Association considers only three parameters for including the MF donkey in the studbook, as the height at withers, the thorax circumference, and the shin circumference. Our study found the mean values for these three parameters are highly representative of the population, with minimal dispersion up to a maximum of 10% across observed target measurements in both genders. This indicates the standard of the MF donkey has been maintained, while additional measurements suggest certain traits within the population denote uniformity. Sexual dimorphism was evident with females presenting with a greater distribution in angular measurements than males.

**Abstract:**

The Martina Franca (MF) donkey breed (*Equus asinus*) primarily inhabits the rural areas surrounding the homonymous municipality, as well as neighboring municipalities in the provinces of Bari and Brindisi, all located in the Apulia region of Italy. The objective of this study was to assess the current phenotype through the evaluation of 27 morphologic measurements. The study was conducted on 73 female and 18 male breeding animals from eight different herds located in central and southern Italy. Statistical analysis was performed in order to demonstrate statistical differences between males and females, as well as sexual dimorphism and uniformity of all measurements in both genders. The results demonstrated that the mean of three parameters used for the evaluation of an MF donkey (height at withers, circumference of thorax, and shin circumference) are highly representative of the population, with minimal dispersion $\hat{C_{v}}$ = 0.05–0.06 up to a maximum of 10%, as evidenced by relatively low standard deviations across observed measurements in both genders. Regarding sexual dimorphism, a statistically significant difference was found between males and females. This dimorphism is linked to reproductive activity and is useful during gestation. Overall, our findings suggest that the MF donkey phenotype has been largely preserved over time with high uniformity in males, slight inhomogeneity in the female population, and less variability in both genders.

## 1. Introduction

**Figure 1:**
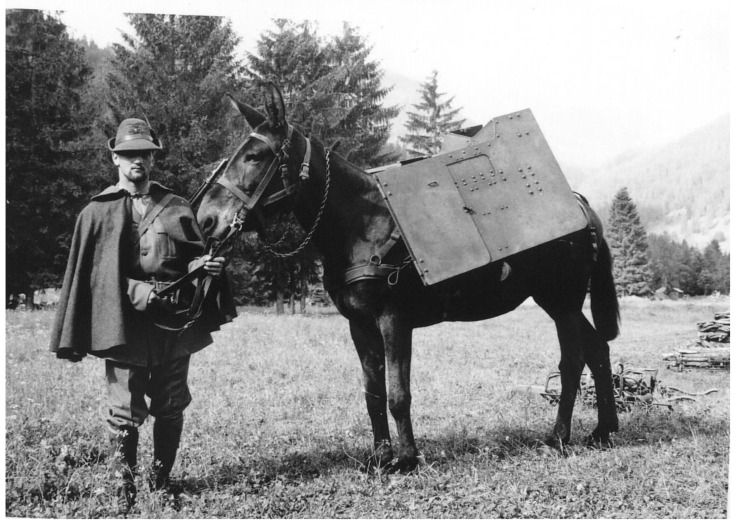
Martina Franca mule of the Italian army used for transporting armaments (picture owned by Augusto Carluccio).

The natural history of the Martina Franca (MF) donkey (*Equus asinus*) [1] has garnered considerable interest due to the escalating imperative to conserve genetic biodiversity [2], and the perceived endangerment of the three primary autochthonous Italian breeds: the MF, Ragusano, and Pantesco donkeys [3]. During the Spanish rule in the Apulia region of Italy, native donkeys were crossed with Catalan donkeys, giving rise to the MF breed [4]. These donkeys are characterized by their tall stature, exceptional robustness, and good temperament. At the beginning of the last century, due to its remarkable features, the MF donkey was exported to countries such as France, Germany, Poland, Slovakia, Hungary, Greece, and even South American countries like Brazil and Argentina [5]. Traditionally, MF donkeys were used during colonization for transporting goods (both for towing and as pack animals), for raising livestock, and occasionally for meat. They were also crucial for mule production using mares from the Murgese horse (*Equus caballus*) breed, which were highly prized in Italy and abroad [6]. In Italy, mules were employed as working animals during the World War I for the transportation of cannons (Figure 1) [7], people, and provisions. They were also used in agriculture, particularly in mountainous areas, until the advent of mechanization. Agricultural mechanization led to the near-complete disappearance of mule populations. However, the utility of mules as working animals in specific environmental contexts, such as marginal mountain agricultural works and wood transportation within national parks, has been re-evaluated recently.

Global investigations into domestic donkeys have not only raised concerns regarding the individual endangerment of donkey breeds but also the species as a whole [8]. Consequently, methodologies facilitating comprehension of the evolutionary trajectories of diverse donkey populations over time, prognostic trends, and the factors influencing such trends assume paramount significance in endeavors aimed at preserving and rejuvenating these populations from their intricate predicaments. The onset of mechanization has precipitated a substantial demographic decline, concurrent with population fragmentation and heightened consanguinity [9,10,11]. Nevertheless, in recent years, the MF donkey has witnessed a resurgence of interest owing to the exploration of alternative applications such as in equine-assisted therapy and the utilization of its milk in cosmetics and infant nutrition. The most recent demo-genetic appraisal of MF genealogical records dates back several decades, when the registered population numbered fewer than 1000 individuals. Historically prized for its unusual stature, the MF donkey was particularly esteemed for mule production; however, with the displacement of draft animal power by agricultural machinery following World War II, mule production gradually waned. The establishment of the MF Donkey and Murgese Horse Breeders’ Association in 1948 and the subsequent founding of the Center for the Conservation and Safeguarding of the MF Donkey by the Apulian regional government in 1981 (Decision No. 12414/81) at the Russoli Farmhouse near Martina Franca reflect concerted efforts towards preservation. The Regional Livestock Management Office, in collaboration with MF donkey breeders and research entities such as the University of Teramo, has been instrumental in altering the population’s numerical trajectory [3]. The MF donkey fulfills many of the criteria deemed imperative for implementing conservation measures aimed at safeguarding specific species or breeds [6]. Its historical significance and ecological role within the agrosilvopastoral system of the Murgia hills are undisputed [12]. Evident in its distinctive morphological characteristics, the MF donkey’s genetic uniqueness is attributed to its adaptive traits essential for combating endemic pathogens prevalent in Apulia. Consequently, this study endeavors to evaluate the phenotype of contemporary MF donkeys through an accurate analysis of obtained data in order to assess the maintenance of the MF standard during last decades.

In particular, we aimed to determine statistical differences between males and females, as well as sexual dimorphism and uniformity of all measurements in both genders. Finally, we compared our data with vis-à-vis archival data from 1930 [13] through an analysis of all 27 morphometric measurements.

## 2. Materials and Methods

### 2.1. The Traditional Breeding Habitat 

**Figure 2:**
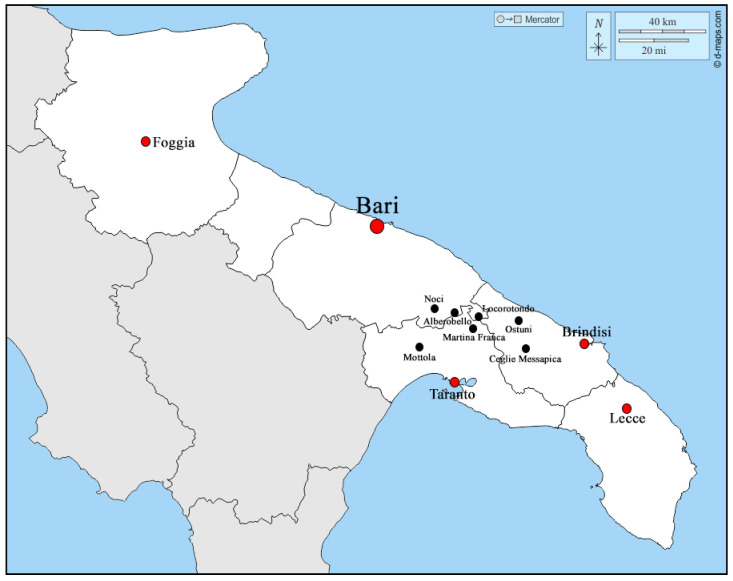
Localities within the provinces of Taranto, Bari, and Brindisi where the Martina Franca donkeys are bred.

Apulia boasts diverse topography encompassing coastlines, woodlands, plains, and hills, with the Murgia hill area constituting a vast calcareous plateau situated at its geographical center. The unique soil composition and climatic conditions of the Murgia hills have historically rendered this region particularly conducive to the breeding of large animals. This association dates back to antiquity, and the rural environs surrounding the town of Martina Franca have emerged as traditional breeding grounds for two notable indigenous equine breeds: the Murgese horse and the MF donkey. The Martina Franca donkey breed primarily inhabits the rural peripheries surrounding its namesake municipality, as well as locales such as Mottola and Massafra within the Taranto province. Additionally, breeding activities extend to neighboring municipalities, including Locorotondo in the Bari province, and Cisternino, Ceglie Messapica, and Ostuni in the Brindisi province. Furthermore, albeit to a limited extent, the breed is found in the elevated terrains of Mottola and Massafra in the province of Taranto, as well as in the municipalities of Alberobello and Noci in the Bari province, all situated within the Apulia region of Italy (Figure 2). 

### 2.2. The Breed 

**Figure 3:**
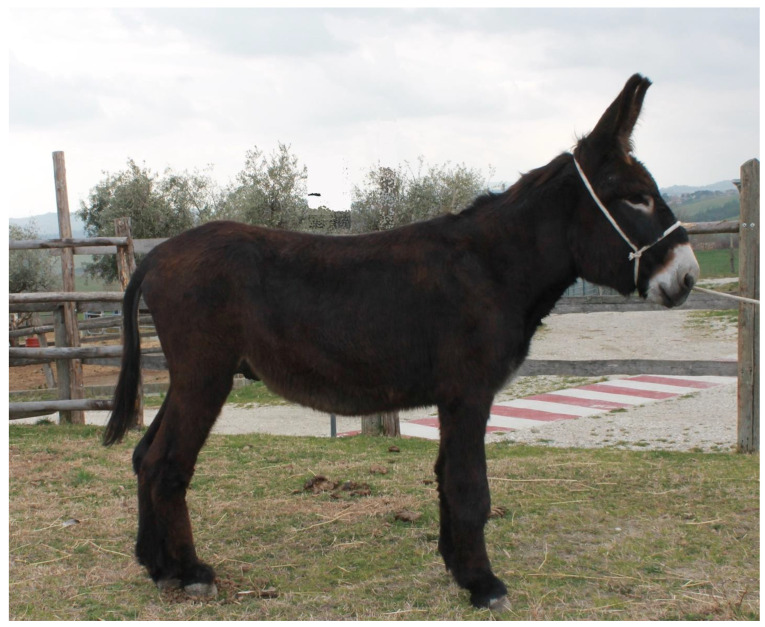
“Nume”, a Martina Franca stallion bred at University of Teramo and included in this study.

**Figure 4:**
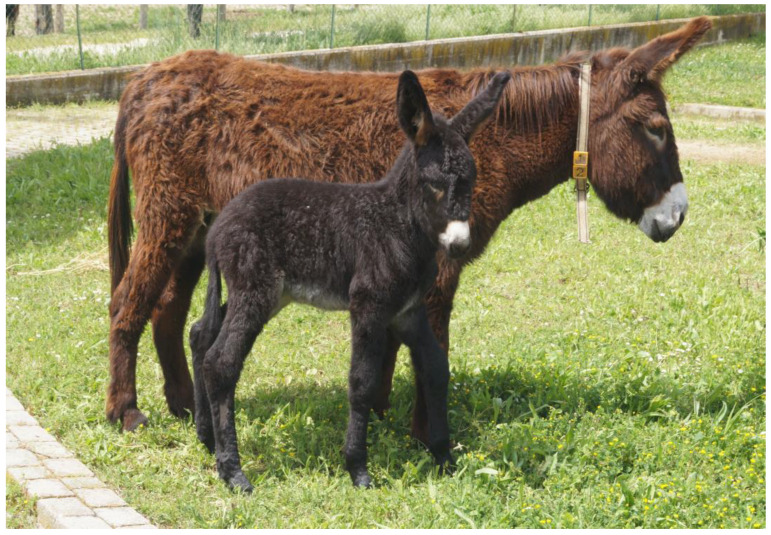
“Alice”, a Martina Franca mare with her foal bred at University of Teramo and included in this study.

The MF donkey is a mesomorphic equine breed characterized by a minimum height at the withers of 135 cm or more in males and 127 cm in females. The minimum chest circumference is 145 cm in males and 140 cm in females, while the minimum circumference of the cannon bone is 19 cm in males and 17 cm in females. This breed displays a dark bay coat color with a dorsal stripe. The abdomen, inner thighs, and muzzle exhibit a gray hue, while the muzzle and eye sockets are surrounded by a reddish halo. The tongue and nasal mucosa are pink. The anus, vulva, scrotum, and prepuce are dark. In particular, the head should be proportionate, with a broad, flat forehead and long, straight ears that are wide at the base, well-attached, mobile, and heavily haired internally. The neck is muscular, with a broad base, particularly prominent in males. The chest is broad and muscular, with well-attached and appropriately inclined shoulders. The thorax is well-developed and deep. The dorsal-lumbar region is straight or slightly saddled, long, muscular, wide, and well-attached. The croup is round, broad, muscular, and well-attached. The tail is well-attached and rich in hair. The limbs are sturdy, with muscular arms and forearms, short pasterns, wide and thick joints, and are free from defects (Figure 3 and Figure 4). The hooves are regular, straight, defect-free, solid, with a sturdy hoof wall, preferably wide. Overall, the donkey should exhibit good development and harmony of form, robust conformation, and proportional diameters. The temperament is lively in males and docile in females [14].

### 2.3. Morphometric Measurements 

**Figure 5:**
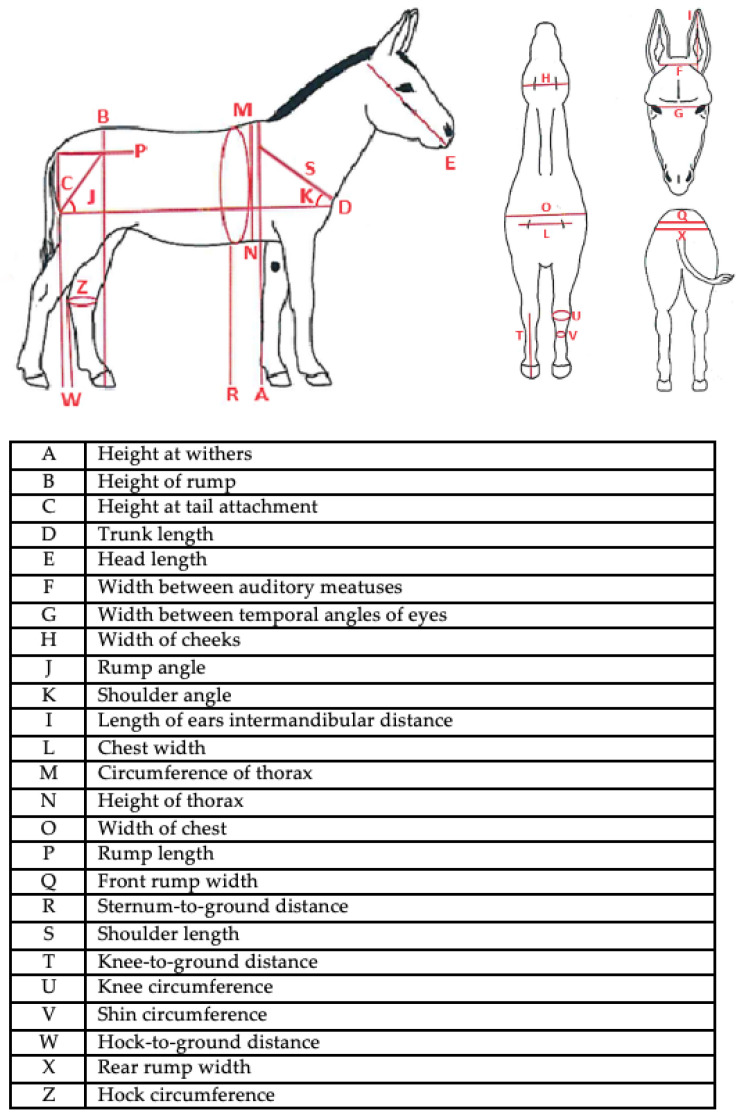
Representation of anatomical regions where measurements were performed using tape measure, Hauptner’s hippometer, and digital animal scales.

Ninety-one MF donkeys (73 female and 18 male breeding animals were included in this study from eight official breeding stations of central and southern of Italy. In detail, all measurements were performed during a clinical visit in the presence of the veterinarian in accordance with the standards for care and protection of animals used for scientific purposes, Directive 2010/63/EU. The 27 measurements included height at withers, height of rump, height at tail attachment, trunk length, head length, width between auditory meatuses, width between temporal angles of eyes, width of cheeks, intermandibular distance, length of ears, width of chest, circumference of thorax, height of thorax, breast width, chest length, rump length, rump angle, front rump width, rear rump width, sternum-to-ground distance, shoulder length, shoulder angle, knee-to-ground distance, knee circumference, shin circumference, hock-to-ground distance, hock circumference, body weight, and the body condition score (BCS) (Figure 5). Measurements were taken using a tape measure, Hauptner’s hippometer, and digital animal scales. Each measurement was replicated three times and performed by the same veterinarian throughout the study to minimize operator error.

### 2.4. Statistical Analysis

The statistical analysis employed several tests to assess the significance of the results. Uniformity of measurements between males and females was evaluated by calculating the estimated coefficient of variation ($\hat{C_{v}}$). Sexual dimorphism was assessed using the multiple unpaired t test two-stage step-up method [15]. 

Pearson’s r correlation matrix was used to determine the uniformity of body measurements. The analysis was conducted using Prism Version 10.2.3—GraphPad Software, LLC (www.graphpad.com, accessed on 25 June 2024) [16,17]. 

## 3. Results

The morphological assessment results of the MF donkey are presented in Table 1 and Table 2 (Findings A), detailing the measurements of 73 females and 18 males as mean and median values, alongside their standard deviations, coefficients of variation, minimum and maximum values, and interquartile ranges. 

To be included in the studbook, the MF Donkey and Murgese Horse Breeders’ Association considers three parameters: height at withers, thorax circumference, and shin circumference. Our study found that the mean values for these three parameters are highly representative of the population, with minimal dispersion ($\hat{C_{v}}\;$= 0.05–0.06) up to a maximum of 10% across observed target measurements in both genders. This indicates that the standard of the MF donkey is maintained, unlike in other studies on the Catalan donkey [18,19]. Other measurements in our study suggest that the phenotypic state of the population meets the requirements, affirming accurate selection of the MF donkey over recent decades. However, angular measurements showed greater dispersion ($\hat{C_{v}}$ = 0.16–0.28 for females and $\hat{C_{v}}$ = 0.19–0.37 for males), as shown in Table 1 and Table 2. 

Regarding sexual dimorphism, a statistically significant difference was found between males and females (Table 3). Males exhibited higher withers height and longer trunk length, whereas females showed a wider rear rump width and a higher croup angle. This dimorphism is linked to reproductive activity and is useful during gestation and parturition. 

**Figure 6:**
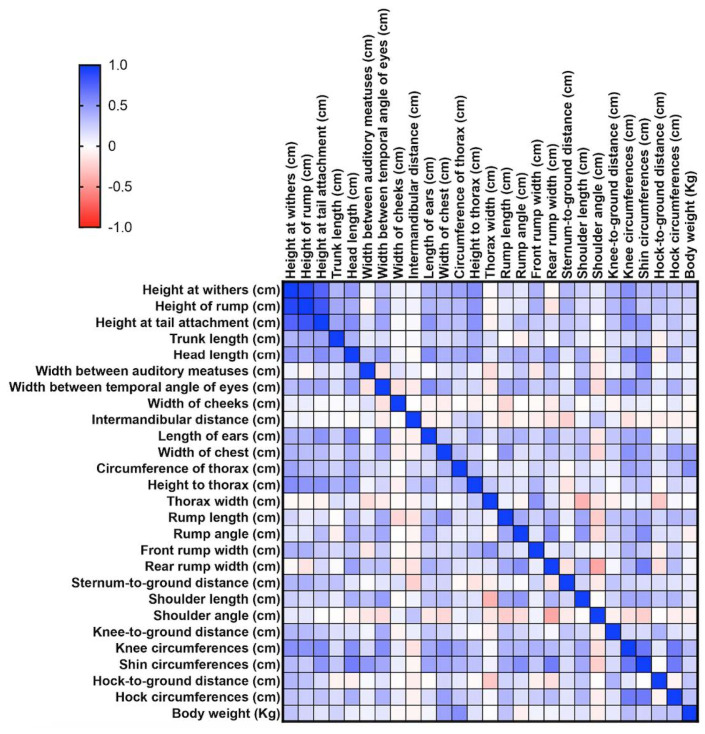
Pearson r correlation matrix for females.

**Figure 7:**
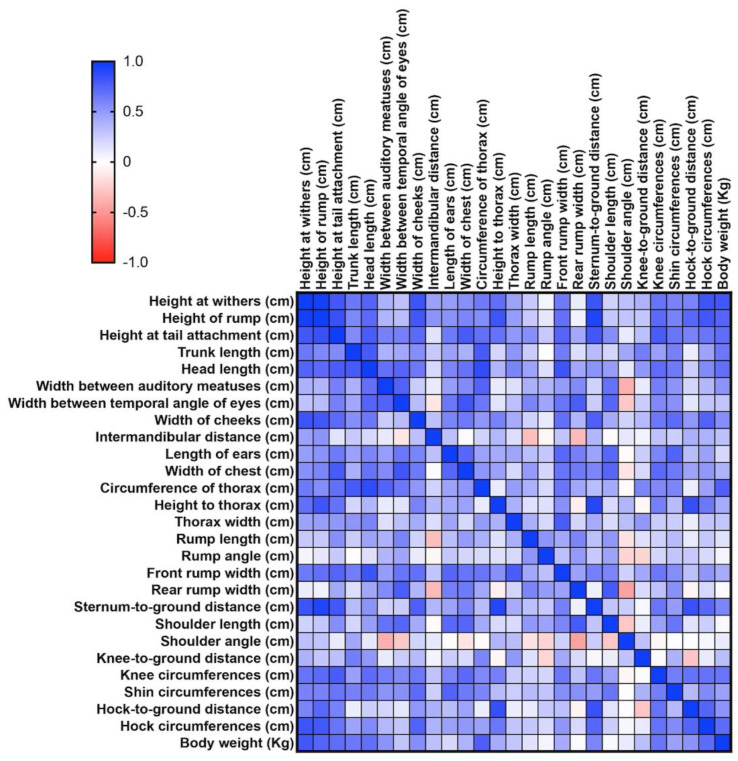
Pearson r correlation matrix for males.

Data distribution was notably uniform, with most Pearson r correlation matrix values between 0.5 and 1, indicating good harmony in the morphometric ratios of the male subjects (Figure 6). We found 166 positive correlations in males (*p* ≤ 0.05), of which 140 with r > 0.5; while 128 correlations were positive (*p* ≤ 0.05) in females, of which 26 were r > 0.5. Specific measurements, such as withers height, rump height, and tail attachment height, showed an *r* tending to 1, between withers height, hock circumference, and body weight. For females, only the three height measurements showed high correlation, while other measurements demonstrated lowest harmony (Figure 7). This disharmony in females is likely due to less stringent selection by breeders compared to stallions.

**Figure 8:**
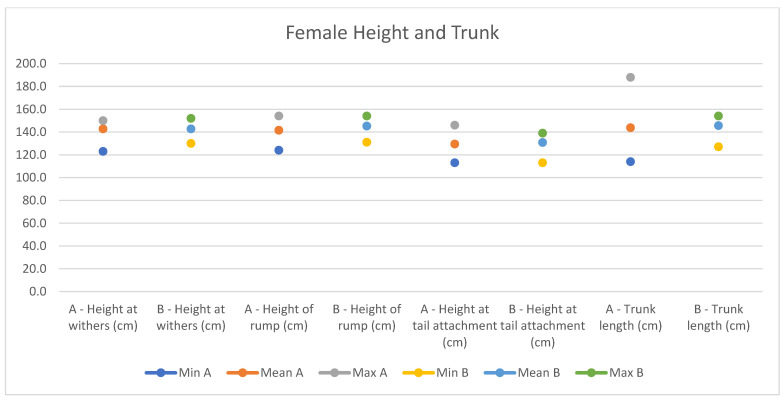
Female measurements of the height and trunk (min, mean, and max) of our research (A) compared with Montanaro’s study (B).

**Figure 9:**
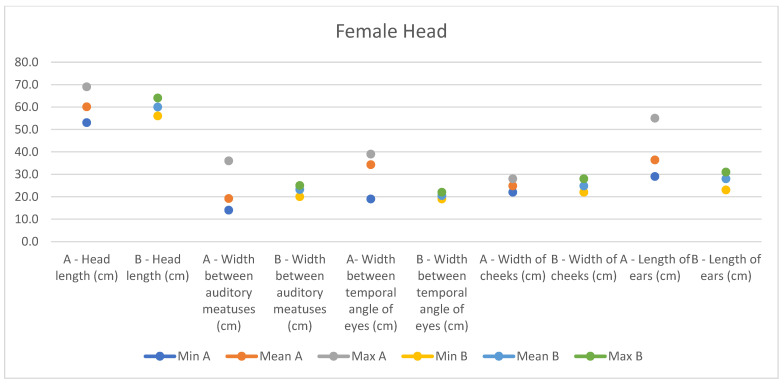
Female measurements of the head (min, mean, and max) of our research (A) compared with Montanaro’s study (B).

**Figure 10:**
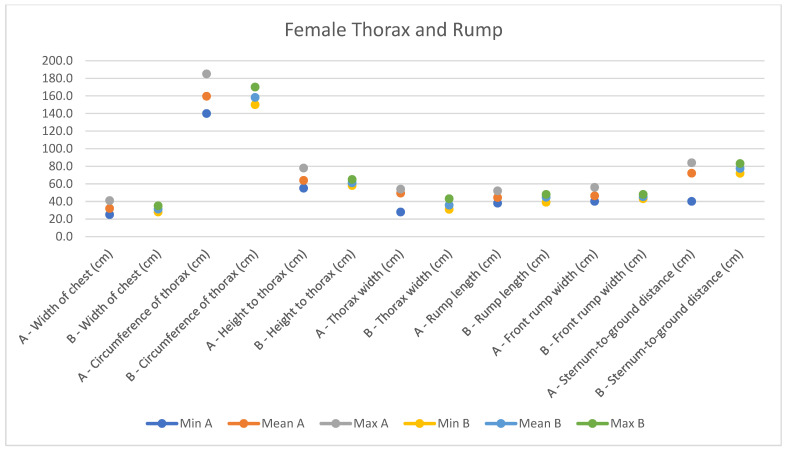
Female measurements of the thorax and rump (min, mean, and max) of our research (A) compared with Montanaro’s study (B).

**Figure 11:**
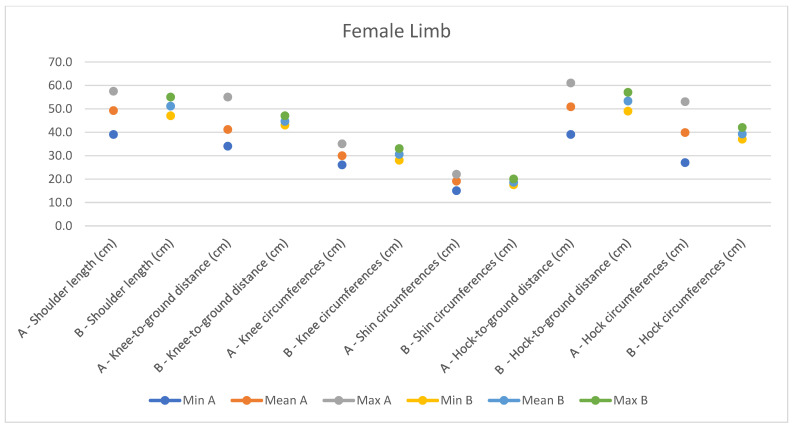
Female measurements of the limb (min, mean, and max) of our research (A) compared with Montanaro’s study (B).

**Figure 12:**
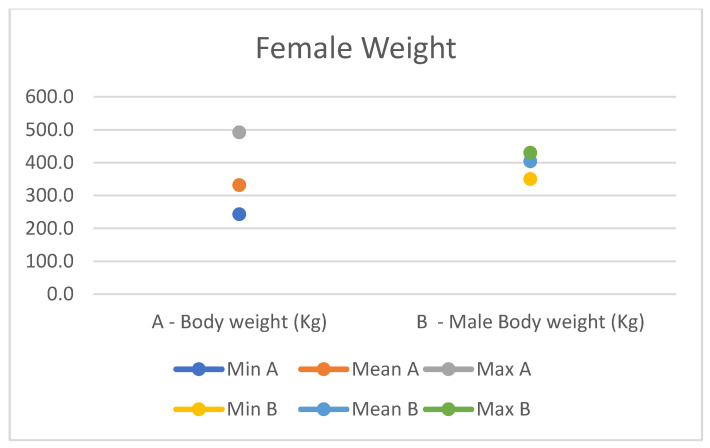
Female measurement of the weight (min, mean, and max) of our research (A) compared with Montanaro’s study (B).

**Figure 13:**
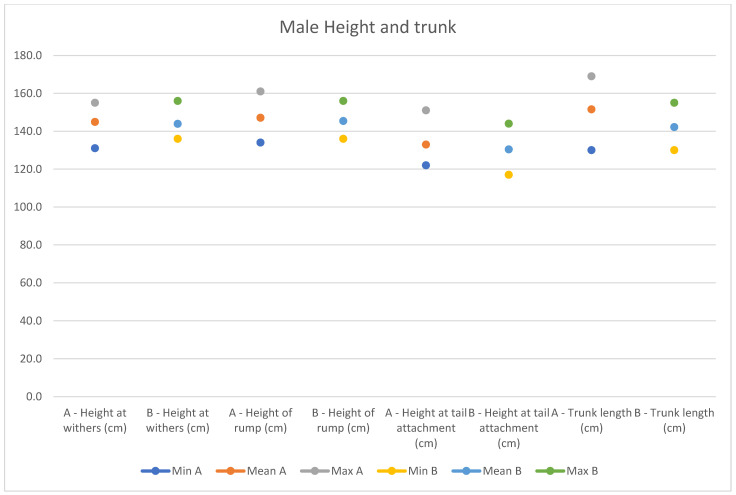
Male measurements of the height and trunk (min, mean, and max) of our research (A) compared with Montanaro’s study (B).

**Figure 14:**
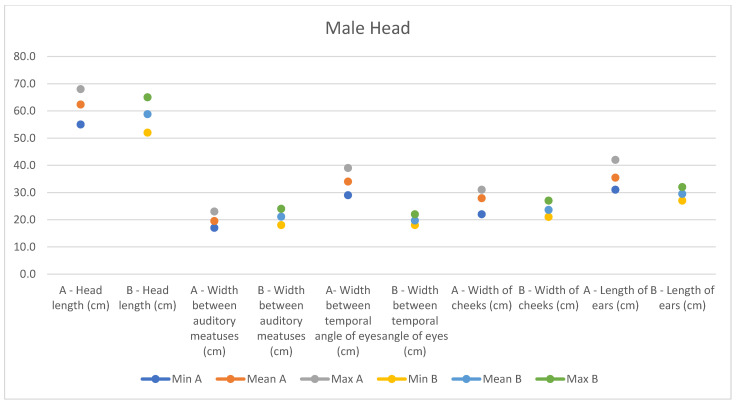
Male measurements of the head (min, mean, and max) of our research (A) compared with Montanaro’s study (B).

**Figure 15:**
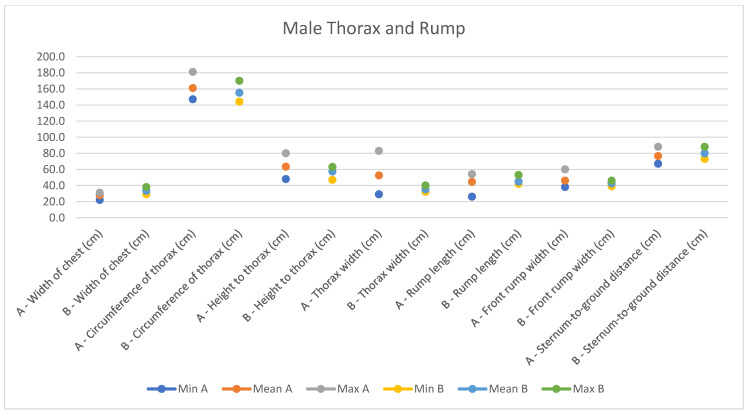
Male measurements of the thorax and rump (min, mean, and max) of our research (A) compared with Montanaro’s study (B).

**Figure 16:**
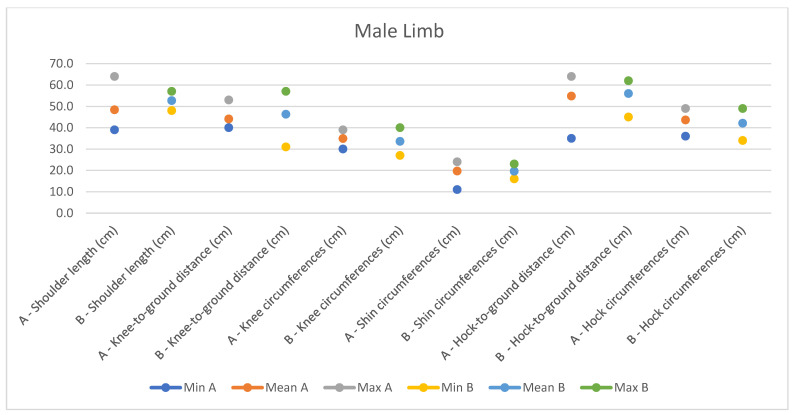
Male measurements of the limb (min, mean, and max) of our research (A) compared with Montanaro’s study (B).

**Figure 17:**
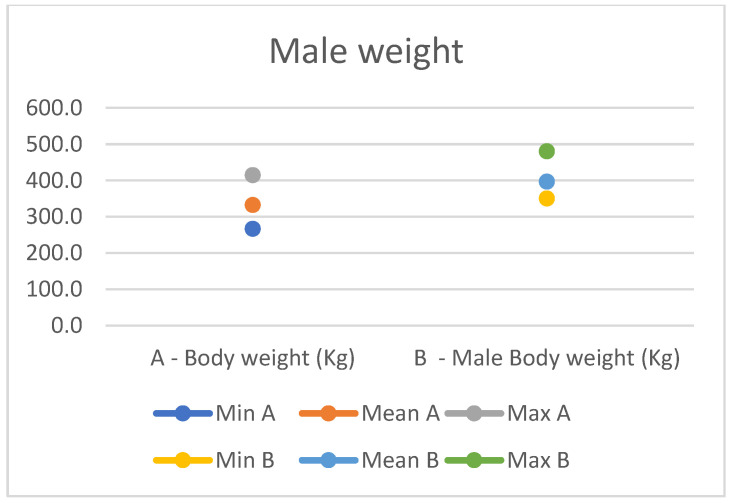
Male measurement of the weight (min, mean, and max) of our research (A) compared with Montanaro’s study (B).

In light of this, we compared our data with those compiled by Montanaro [13], with Table 4 and Table 5 (Findings B) representing the sole literature study on MF donkeys permitting a direct comparison. Regrettably, Montanaro provided only the averages $(\bar{x}$) and the maximum and minimum values (Min/Max) without the standard deviations (s) for each observed parameter (Figure 8, Figure 9, Figure 10, Figure 11, Figure 12, Figure 13, Figure 14, Figure 15, Figure 16 and Figure 17). Consequently, the absence of raw data precluded evaluations of statistical significance, which would have offered precise insights into potential morphological variations occurring in this breed since 1930. Nonetheless, trend indications can be observed from the extensive array of measurements (27 morphological parameters) conducted in our study. For females, comparisons of mean height at withers, rump height, sternum-to-ground distance, trunk length, and knee-to-ground distance revealed a tendency towards a diminished stature. Additionally, a decrease in average weight was noted. Noteworthy alterations in head morphology included increased width between temporal angles of the eyes and decreased width between auditory meatus, alongside elongation of the ears. Conversely, for males, stature appears increased, potentially accompanied by increased trunk length, thoracic circumference, thoracic width, and a decrease in sternum-to-ground distance. Similar to females, male head morphology exhibited lengthening, widening between temporal angles of the eyes, cheek width, and ear length, along with a decrease in body weight.

## 4. Discussion

Based on the morphological measurements required by the MF Donkey and Murgese Horse Breeders’ Association for studbook inclusion (height at withers, thorax circumference, and shin circumference), we conducted a phenotypic evaluation of the MF donkey, measuring 27 parameters. We demonstrated that the mean of three parameters used for the evaluation of an MF donkey are highly representative of the population. We also performed an accurate analysis in order to highlight significant differences between males and females. Males exhibited higher withers height and longer trunk length, whereas females showed a wider rear rump width and a higher croup angle. We supposed that dimorphism was linked to reproductive activity and it could be useful during gestation.

Other measurements in our study suggest that the phenotypic state of the population meets the requirements, affirming accurate selection of the MF donkey over recent decades. In detail, results indicated that the population showed uniformity in both genders, and harmonicity was more relevant in males, while angular measurements showed greater dispersion especially in females.

Comparing our results with those of Montanaro’s, we showed a decrease in auditory meatus width, sternum-to-ground distance, knee-to-ground distance, and body weight in females, with an increase in the width between temporal angles of the eyes, ear length, and thorax width. In males, trunk length, head length, thorax circumference, and various other measurements increased, alongside a decrease in sternum-to-ground distance and body weight. Overall, our findings suggest that the MF donkey phenotype has been largely preserved over time with high uniformity, slight disharmony in the female population, and less variability in both genders. 

These findings suggest a probable shift in the practices of MF donkey breeders over the decades, indicating that the donkey has assumed a significant role beyond mere towing activities, contributing to social development. In accordance with the Convention on Biological Diversity [20], in situ conservation emerges as a primary imperative for the preservation of livestock breeds [21,22], with economic sustainability being a fundamental prerequisite. Currently, economic viability does not appear to pose a significant challenge for the MF donkey. The growing demand for the MF donkey, particularly in agritourism and educational farms across Italy, where the breed’s attributes are highly valued, reflects a positive trajectory [23], emphasizing the crucial role certain breeds play in nurturing and perpetuating local customs [24]. Given its longstanding presence and integral role in the agricultural landscape, especially within the Murgia region, the MF donkey has garnered increased attention from local communities aiming to revive shared Apulian cultural and traditional values. However, despite alleviating concerns regarding breeder abandonment, the primary conservation challenge lies in ensuring its biological sustainability. A comprehensive understanding of the breed’s origin, historical trajectory, and evolution is essential for formulating sustainable conservation and utilization strategies [25,26,27]. The breed’s renewed significance has led to new market opportunities, such as the production of donkey milk for pediatric use and the cosmetics industry. The Martina Franca donkeys are also involved in educational, assisted, and therapeutic activities with animals (onotherapy) [28,29,30].

## 5. Conclusions

The MF donkey, named after the Apulian town of Martina Franca in the Murgia area of Taranto province, is the world’s most renowned donkey breed. Its size has facilitated its export worldwide, and it is highly valued for producing exceptional mules and improving other donkey populations. 

The MF donkey’s stature and morphological structure make it particularly suitable for working, trekking, and mule production, which has been appreciated in Italy and abroad. Following World War II, the breed’s numbers declined due to increased agricultural mechanization and a reduction in mule production. By the early 1980s, the future of the MF donkey breed was in jeopardy, with many breeding nuclei lost. However, since the 1990s, interest in the breed has surged. 

Fortunately, a promising future awaits the MF donkey. Increased awareness and governmental sensitivity within the Apulian regional administration led to the enactment of comprehensive regional legislation on 13 May 2009. This legislation encompasses the protection and conservation oriented for utilization of indigenous Apulian agricultural and forest resources, including both natural and cultivated plant species, as well as farm animals, such as the MF donkey.

Over the past 90 years, despite a reduction in the number of subjects due to mechanization in agriculture and the advent of new means of transport, the Martina Franca donkey has retained its phenotypic characteristics. However, during a critical phase of breed conservation, which faced the risk of extinction, it was not possible to make precise selections in the breeding of mares, even those with mediocre phenotypic characteristics were included in reproduction plans. Of fundamental importance is the data on sexual dimorphism, specifically regarding the angle of the pelvis in females compared to males. This feature facilitates foal birth, particularly in individuals with significantly reduced stature and shorter trunk length. Easier calving correlates with a lower percentage of dystocia and the consequent birth of viable foals, contributing to the maintenance of the breed.

In light of our findings, it is recommended that the MF Donkey and Murgese Horse Breeders’ Association consider this study to refine the parameters used when evaluating 30-month-old subjects for registration in the studbook. This consideration is especially crucial for females, as the population’s reduced uniformity necessitates careful selection to enhance typicality, similar to the standards applied to males. Additionally, the excellent sexual dimorphisms should not be overlooked, as they significantly contribute to the functionality of these remarkable animals.

## Figures and Tables

**Table 1:** Results of body measurements of this study for female Martina Franca donkeys (A) (*n* = number of animals observed; $\bar{x}$ = mean; s = standard deviation; $\hat{C_{v}}$ = estimated coefficient of variation, Min/Max = minimum–maximum observed values; median, q_1_ = lower quartile; q_3_ = upper quartile).

FEMALE Morphometric Measure	Findings (A)							
	*n*	$\bar{x}$	s	$\hat{C_{v}}$	Min/Max	Median	q_1_	q_3_
Height at withers (cm)	73	137.59	6.08	0.04	123–150	138	133	142
Height of rump (cm)	73	141.35	6.41	0.05	124–154	142	136.5	154
Height at tail attachment (cm)	73	129.41	7.14	0.06	113–146	129	124.5	135.5
Trunk length (cm)	73	143.75	9.49	0.07	114–188	143	138.5	148
Head length (cm)	73	60.06	3.79	0.06	53–69	60	57.5	62.00
Width between auditory meatuses (cm)	73	19.18	2.99	0.16	14–36	19	17	21
Width between temporal angle of eyes (cm)	73	34.29	3.29	0.10	19–39	35	33	36
Width of cheeks (cm)	73	24.81	1.79	0.07	22–28	25	23.5	27
Intermandibular distance (cm)	73	12.73	1.28	0.10	10–16	13	12	14
Length of ears (cm)	73	36.40	4.10	0.11	29–55	36	34	39
Width of chest (cm)	73	31.97	3.37	0.11	25–41	32	29	34
Circumference of thorax (cm)	73	158.19	15.81	0.06	140–185	160	151.5	185
Height to thorax (cm)	73	63.87	4.60	0.07	55–78	63	60.5	67
Thorax width (cm)	73	49.47	10.09	0.20	28–54	50	46	54
Rump length (cm)	73	44.35	3.92	0.09	38–52	44	42	47.5
Rump angle (cm)	73	31.37	8.75	0.28	15–50	30	25	40
Front rump width (cm)	73	46.32	2.98	0.06	40–56	46	44	48
Rear rump width (cm)	73	25.64	6.04	0.24	12–37	28	19.5	30
Sternum-to-ground distance (cm)	73	72.03	6.77	0.09	40–84	72	69.5	75
Shoulder length (cm)	73	49.23	4.00	0.08	39–57.5	50	47	52
Shoulder angle (cm)	73	52.66	8.50	0.16	40–77	50	45	60
Knee-to-ground distance (cm)	73	41.13	3.11	0.08	34–55	41	39.75	43
Knee circumferences (cm)	73	29.95	2.12	0.07	26–35	30	28	31.25
Shin circumferences (cm)	73	19.07	1.92	0.10	15–22	19	18	21
Hock-to-ground distance (cm)	73	50.83	4.59	0.09	39–61	50	48	54.75
Hock circumferences (cm)	73	39.84	4.07	0.10	27–53	38	36	41
Body weight (Kg)	73	331.42	49.36	0.15	243–492	323	299	365.5
Body condition score (BCS)	73				1–4	3	3	4

**Table 2:** Results of body measurements of this study for male Martina Franca donkeys (A) (*n* = number of animals observed; $\bar{x}$ = mean; s = standard deviation; $\hat{C_{v}}$ = estimated coefficient of variation, Min/Max = minimum–maximum observed values; median, q_1_ = lower quartile; q_3_ = upper quartile).

MALE Morphometric Measure	Findings (A)							
	*n*	$\bar{x}$	s	$\hat{C_{v}}$	Min/Max	Median	q_1_	q_3_
Height at withers (cm)	18	144.94	7.17	0.05	131–155	143.5	141	153
Height of rump (cm)	18	147.11	7.39	0.05	134–161	145	143	154
Height at tail attachment (cm)	18	132.94	8.20	0.06	122–151	133.5	125.8	139
Trunk length (cm)	18	151.56	9.44	0.06	130–169	151.5	145	160
Head length (cm)	18	62.33	3.58	0.06	55–68	61.5	60	65.25
Width between auditory meatuses (cm)	18	19.50	2.01	0.10	17–23	19	18	21.25
Width between temporal angle of eyes (cm)	18	34.00	3.36	0.10	29–39	34.5	30.75	36.5
Width of cheeks (cm)	18	27.89	2.40	0.09	22–31	27	26.75	30.25
Intermandibular distance (cm)	18	13.92	1.66	0.12	9–17	14	13	15
Length of ears (cm)	18	35.44	3.31	0.09	31–42	35	33.5	37.25
Width of chest (cm)	18	33.78	3.66	0.11	28–41	33	33	38
Circumference of thorax (cm)	18	161.50	9.99	0.06	147–181	161.5	152	169.3
Height to thorax (cm)	18	63.39	6.43	0.10	48–80	63	60.50	67
Thorax width (cm)	18	52.50	10.41	0.20	29–83	51.5	48.75	54.25
Rump length (cm)	18	44.44	6.36	0.14	26–54	45	41.75	48.25
Rump angle (cm)	18	23.33	8.74	0.37	15–45	20	18.75	26.25
Front rump width (cm)	18	45.94	4.63	0.10	38–60	45	43.75	48
Rear rump width (cm)	18	19.78	6.62	0.33	12–32	17.5	14	23.75
Sternum-to-ground distance (cm)	18	76.61	5.01	0.07	67–88	75.5	73.75	79.5
Shoulder length (cm)	18	48.39	7.15	0.15	39–64	46.5	43	53.5
Shoulder angle (cm)	18	55.56	10.42	0.19	30–70	60	48.75	60
Knee-to-ground distance (cm)	18	44.11	3.27	0.07	40–53	44	41.75	46
Knee circumferences (cm)	18	34.92	2.78	0.08	30–39	34.5	32.75	37.25
Shin circumferences (cm)	18	19.67	3.56	0.18	11–24	20	19	22
Hock-to-ground distance (cm)	18	54.83	6.03	0.11	35–64	56	51	59.25
Hock circumferences (cm)	18	43.61	3.27	0.07	36–49	45	41.5	46
Body weight (Kg)	18	331.72	45.14	0.14	266–414	320	294	377.3
Body condition score (BCS)	18		0		2–4	3	3	3

**Table 3:** Results of comparison between the mean for statistically significant measurements (*p* ≤ 0.05) for both genders.

	*p*-Value	Mean of Males	Mean of Females	Difference	SE of Difference	t Ratio	df	q Value
Knee circumferences (cm)	<0.000001	34.92	29.95	4.97	0.595	8.353	89	<0.000001
Width of cheeks (cm)	<0.000001	27.89	24.81	3.08	0.5057	6.091	89	<0.000001
Hock circumferences (cm)	0.000013	43.61	38.84	4.77	1.034	4.612	89	0.000067
Height at withers (cm)	0.000027	144.9	137.6	7.35	1.659	4.431	89	0.000101
Rear rump width (cm)	0.000493	19.78	25.64	−5.86	1.62	3.618	89	0.001352
Knee-to-ground distance (cm)	0.000536	44.11	41.14	2.97	0.8266	3.593	89	0.001352
Rump angle (cm)	0.000748	23.33	31.37	−8.04	2.302	3.492	89	0.001618
Intermandibular distance (cm)	0.001294	13.92	12.73	1.19	0.3581	3.323	89	0.002269
Height of rump (cm)	0.001364	147.1	141.4	5.75	1.739	3.306	89	0.002269
Sternum-to-ground distance (cm)	0.001498	76.61	71.03	5.58	1.703	3.277	89	0.002269
Trunk length (cm)	0.002362	151.6	143.8	7.81	2.495	3.13	89	0.003253
Hock-to-ground distance (cm)	0.002912	54.83	50.83	4	1.307	3.062	89	0.003676

**Table 4:** Results of the study for female Martina Franca donkeys (A) compared with Montanaro’s findings (B). (*n* = number of animals observed; $\bar{x}$ = mean; Min/Max = minimum–maximum observed values).

FEMALES Morphometric Measure (cm)	Findings (A)			Montanaro Findings (B)		
	*n*	$\bar{x}$	Min/max	*n*	$\bar{x}$	Min/max
Height at withers (cm)	73	137.59	123–150	10	142.7	130–152
Height of rump (cm)	73	141.35	124–154	10	145.2	131–154
Height at tail attachment (cm)	73	129.41	113–146	10	130.8	113–139
Trunk length (cm)	73	143.75	114–188	10	145.6	127–154
Head length (cm)	73	60.06	53–69	10	60	56–64
Width between auditory meatuses (cm)	73	19.18	14–36	10	23.2	20–25
Width between temporal angle of eyes (cm)	73	34.29	19–39	10	20.5	19–22
Width of cheeks (cm)	73	24.81	22–28	10	24.8	22–28
Intermandibular distance (cm)	73	12.73	10–16	--	--	--
Length of ears (cm)	73	36.40	29–55	9	28	23–31
Width of chest (cm)	73	31.97	25–41	10	31.3	28–35
Circumference of thorax (cm)	73	159.56	140–185	8	158.2	150–170
Height to thorax (cm)	73	63.87	55–78	9	61.1	58–65
Thorax width (cm)	73	49.47	28–54	9	35.7	31–43
Rump length (cm)	73	44.35	38–52	10	44.7	39–48
Rump angle (cm)	73	31.37	15–50	--	--	--
Front rump width (cm)	73	46.32	40–56	10	45.3	43–48
Rear rump width (cm)	73	25.64	12–37	--	--	--
Sternum-to-ground distance (cm)	73	72.03	40–84	9	77.5	72–83
Shoulder length (cm)	73	49.23	39–57.5	9	51.1	47–55
Shoulder angle (cm)	73	52.66	40–77	--	--	--
Knee-to-ground distance (cm)	73	41.13	34–55	8	44.6	43–47
Knee circumferences (cm)	73	29.95	26–35	9	30.5	28–33
Shin circumferences (cm)	73	19.07	15–22	9	18.6	17.5–20
Hock-to-ground distance (cm)	73	50.83	39–61	9	53.3	49–57
Hock circumferences (cm)	73	39.84	27–53	8	39.2	37–42
Body weight (Kg)	73	331.42	243–492	5	404	350–430

**Table 5:** Results of the study for male Martina Franca donkeys (A) compared with Montanaro’s findings (B). (*n* = number of animals observed; $\bar{x}$ = mean; Min/Max = minimum–maximum observed values).

MALES Morphometric Measure (cm)	Findings (A)			Montanaro Findings (B)		
	*n*	$\bar{x}$	Min/max	*n*	$\bar{x}$	Min/max
Height at withers (cm)	18	144.94	131–155	16	143.9	136–156
Height of rump (cm)	18	147.11	134–161	15	145.4	136–156
Height at tail attachment (cm)	18	132.94	122–151	13	130.4	117–144
Trunk length (cm)	18	151.56	130–169	16	142.2	130–155
Head length (cm)	18	62.33	55–68	13	58.8	52–65
Width between auditory meatuses (cm)	18	19.50	17–23	16	21.1	18–24
Width between temporal angle of eyes (cm)	18	34.00	29–39	15	19.7	18–22
Width of cheeks (cm)	18	27.89	22–31	14	23.6	21–27
Intermandibular distance (cm)	18	13.92	9–17	--	--	--
Length of ears (cm)	18	35.44	31–42	15	29.5	27–32
Width of chest (cm)	18	33.78	28–41	16	33.4	29–38
Circumference of thorax (cm)	18	161.50	147–181	16	155.1	144–170
Height to thorax (cm)	18	63.39	48–80	16	57.4	47–63
Thorax width (cm)	18	52.50	29–83	16	35.2	32–40
Rump length (cm)	18	44.44	26–54	16	44.7	42–53
Rump angle (cm)	18	23.33	15–45	--	--	--
Front rump width (cm)	18	45.94	38–60	16	42.7	39–46
Rear rump width (cm)	18	19.78	12–32	--	--	--
Sternum-to-ground distance (cm)	18	76.61	67–88	16	80	73–88
Shoulder length (cm)	18	48.39	39–64	15	52.7	48 -57
Shoulder angle (cm)	18	55.56	30–70	--	--	--
Knee-to-ground distance (cm)	18	44.11	40–53	16	46.3	31–57
Knee circumferences (cm)	18	34.92	30–39	16	33.6	27–40
Shin circumferences (cm)	18	19.67	11–24	16	19.6	16–23
Hock-to-ground distance (cm)	18	54.83	35–64	15	56	45–62
Hock circumferences (cm)	18	43.61	36–49	13	42.1	34–49
Body weight (Kg)	18	331.72	266–414	11	396	350–480

## Data Availability

All the data are provided within the manuscript.
